# Optimization of the GaAs-on-Si Substrate for Microelectromechanical Systems (MEMS) Sensor Application

**DOI:** 10.3390/ma5122917

**Published:** 2012-12-17

**Authors:** Yunbo Shi, Hao Guo, Haiqiao Ni, Chenyang Xue, Zhichuan Niu, Jun Tang, Jun Liu, Wendong Zhang, Jifang He, Mifeng Li, Ying Yu

**Affiliations:** 1 Key Laboratory of Instrumentation Science & Dynamic Measurement Ministry of Education, Taiyuan, Shanxi 030051, China; E-Mails: shiyunbo@nuc.edu.cn (Y.S.); guohaonuc@163.com (H.G.); xuechenyang@nuc.edu.cn (C.X.); tangjun@nuc.edu.cn (J.T.); 2 State Key Laboratory for Superlattices and Microstructures, Institute of semiconductors, Chinese Academy of Sciences, Beijing 100083, China; E-Mails: nihq@semi.ac.cn (H.N.); zhniu@semi.ac.cn (Z.N.); wdzhang@nuc.edu.cn (W.Z.); hejifang@semi.ac.cn (J.H.); limifeng@semi.ac.cn (M.L.); yuying@semi.ac.cn (Y.Y.); 3 School of Mechatronic Engineering, Beijing Institute of Technology, 100081, China

**Keywords:** residual stress, GaAs-on-Si, MEMS sensors

## Abstract

Resonant Tunneling Diodes (RTD) and High Electron Mobility Transistor (HEMT) based on GaAs, as the piezoresistive sensing element, exhibit extremely high sensitivity in the MEMS sensors based on GaAs. To further expand their applications to the fields of MEMS sensors based on Si, we have studied the optimization of the GaAs epitaxy layers on Si wafers. Matching superlattice and strain superlattice were used, and the surface defect density can be improved by two orders of magnitude. Combing with the Raman spectrum, the residual stress was characterized, and it can be concluded from the experimental results that the residual stress can be reduced by 50%, in comparison with the original substrate. This method gives us a solution to optimize the epitaxy GaAs layers on Si substrate, which will also optimize our future process of integration RTD and HEMT based on GaAs on Si substrate for the MEMS sensor applications.

## 1. Introduction

Due to their high electron mobility, low power consumption and photoelectric characteristics, the resonant tunnel diode (RTD) and high electron mobility transistor (HEMT) are widely applied as electronic devices and photoelectric devices. From our previous study, the RTD and HEMT show a high piezoresistive coefficient [1]. They can be used as the sensitive elements of MEMS sensors, which can greatly improve their sensitivity of the MEMS sensors [2]. From the experimental results, it already has been demonstrated that the sensitivity of MEMS that use HEMT is two to three orders of magnitude higher than those using the Si piezorsistive elements [3], and those using RTD are one to two orders of magnitude higher [4,5].

It was also found that the GaAs substrate is fragile and costly, which has limited the application of GaAs and semiconductor devices based on GaAs as the mechanical sensors. Due to this issue, a new method to combine the high sensitivity of RTD or HEMT with the Si substrate is quite important.

In the past 30 years, Si has been well established as a semiconductor material for microelectronics. To expand the application, different technologies have been developed to integrate Si with other semiconductor materials, such as direct GaAs-on-Si epitaxy [6], GaAs-on-Si growth through Ge buffer layers [7], GaAs-on-SOI epitaxy [8], GaAs-on-STO-Si epitaxy [9], GaAs on nanostructure Si substrate [10], GaAs on nanostructure SiO_2_-on-Si [11], Conformal growth method [12], Bonding, and so on.

IBM has used the Ge buffer by growing the GaAs/Si material and making the RMS of material surface less than 1nm. They also have grown GaAs on the 200–300 mm Si substrate [13]. Stanford University has manufactured the Metal-Oxide-Semiconductor Capacitors (MOSCAP). That offers the first step toward future applications involving high- κ Ш-V MOSFETs on the Si substrate [14].

It can be concluded from the literature that the GaAs-on-Si has already been well studied, and some successful applications on the electronic devices have also been reported. The application of the GaAs-on-Si substrates to the MEMS sensors is still lacking in research.

In this paper, we demonstrated a surface optimized method of the GaAs-on-Si substrate, which can be used in the MEMS sensors. The superlattice structure is fabricated on the commercially available Si/GaAs wafer. Using the Raman spectrum, the residual stress was found to be reduced by 50%, in comparison with the original substrate. Meanwhile, the defect density has improved by two orders of magnitude. This method gives us a solution to optimize the epitaxy GaAs layers on the Si substrate, which will also optimize our future process of integration RTD and HEMT for the MEMS sensor.

## 2. Experiments

Commercially available GaAs-on-Si wafers were used and were purchased from the Spire Company, USA. The GaAs layer was directly grown on the 3 inch Si wafers. N^+^-Si wafers with doping concentrations of 5 × 10^16^ cm^−2^ and a thickness of 350 μm were used as the substrate. GaAs epilayers were grown on exactly (100) oriented Si with 4° misorienting toward (111) Si substrate with a thickness of 2 μm. The initial density of the lattice defect of the purchased GaAs/Si wafers is 10^6^ cm^−2^.

**Figure 1:**
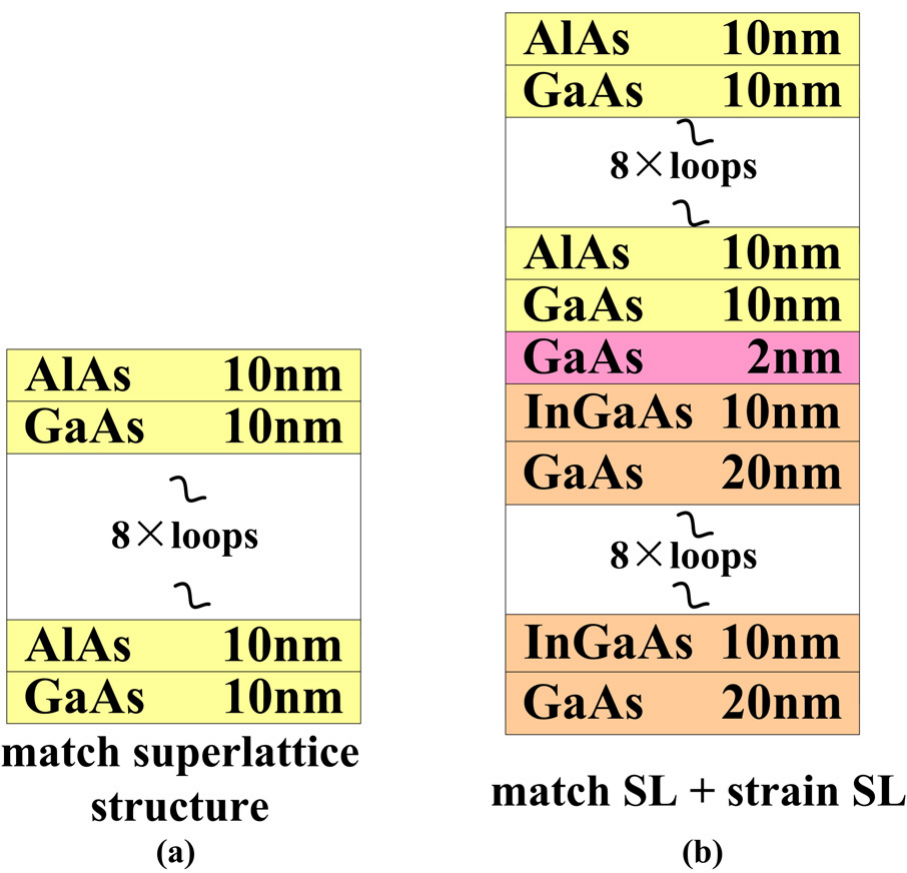
Two different optimization structures.

The optimized epilayers in our experiment were grown using the Molecular beam epitaxy (MBE), Veeco Mod-GEN П (the products of the Veeco Instruments Inc, Somerset, NJ, USA). Two different solutions were used in this paper: AlAs/GaAs superlattice and InGaAs/GaAs strain superlattice (as shown in Figure 1). The surface topography and cross-section of the epilayers were characterized by transmission electron microscopy (TEM, FEI Tecnai G2 F20, the products of the FEI Company, Hillsboro, OR, USA), scanning electron microscopy (SEM, KYKY-1000B, the products of the Chinese academy of sciences, Beijing science instrument development center, Beijing, China) and atomic force microscopy (AFM, Nanonavi Esweep S-II, the products of the SEIKO Co. Ltd, Tokyo, Japan).

The residual stress is studied by the Renishaw Raman Microscope System Invia (the laser line is 514.5 nm; the excitation beam power is 5 mW). The luminescence characteristics of the quantum well (QW) were used by the Fourier Transforms Infrared Spectrometer (Nicolet FTIR760 , the products of the Nicolet Co. Ltd, Appleton, WI, USA). The power was 1 W, and the wavelength was 632.8 nm.

## 3. Results and Discussion

### 3.1. Wafer Test

**Figure 2:**
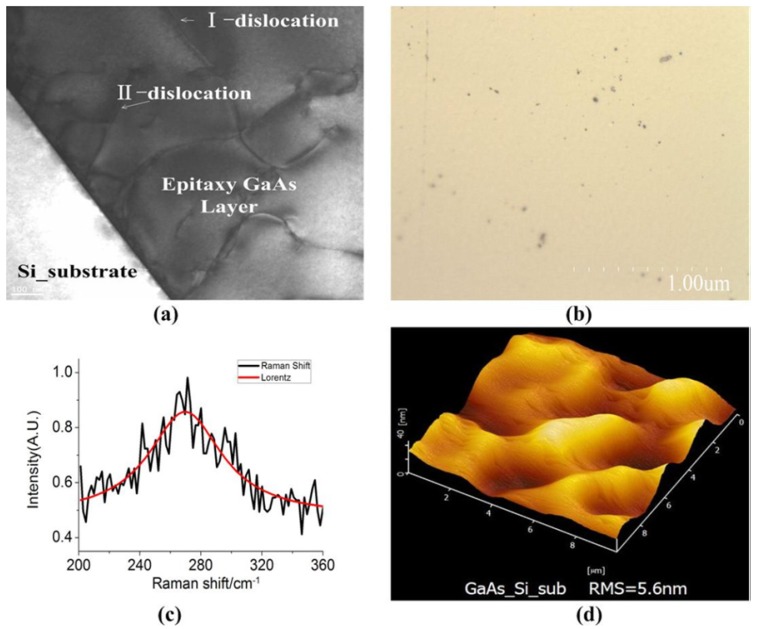
Wafer test: (**a**) the cross-section TEM image; (**b**) the SEM image of etch pit of dislocation; (**c**) Raman spectra; and (**d**) the AFM image.

To have a better understanding of the purchased wafers, we have first quantitatively characterized the defects on the GaAs/Si wafers, as it is shown in Figure 2:

Figure 2a shows the TEM image of the cross-section, which shows the GaAs layer on the Si substrate. The edge dislocation and the screw dislocation at the interface were clearly observed. They extend up to the surface of the epitaxy GaAs layer. This means that there are several dislocations caused by lattice mismatch and thermal mismatch in the GaAs-on-Si wafer. To have a more quantitative characterization of this lattice mismatch, the surface lattice defect was characterized by FESEM. As it is shown in Figure 2b, the wafer has been etched (by HF, H_2_O, AgNO_3_ and CrO_3_ miscible liquids in certain proportion [15]) one minute at 30 °C before the FESEM characterization. The etch pit density (EPD) in the image is 1.84 × 10^6^ cm^−2^. This means the density of lattice defect is 10^6^ cm^−2^.

Raman spectral analysis is the primary technique to test the residual stress of a material. The Raman spectrum was used to calculate the residual stress after the optimization by the super lattice structure. The residual stress acting is best expressed as follow:

(1) σ=−576Δω

where σ is the residual stress and Δω is the wave number shift [16].

Figure 2c shows the Raman spectroscopy image of the wafer using Lorentz fit. The Raman peak value of GaAs single crystal is 267 cm^−1^. The image shows that the peak value of the wafer is 269.72 cm^−1^. The peak of the wafer has a blue shift to the peak of GaAs. The value is 2.72 cm^−1^, and the full width at half maximum (FWHM) is 60.09. The residual stress depends linearly on the wave number shift, as in Equation (1). This means the residual stress in the wafer is compressive stress and the value is 1.57 GPa. The curve also shows that the longitudinal phonon spectroscopy is weak. Because of that, there is the lattice defect, surface roughness and inner strain, which can enhance the probability of impurity scattering and weaken the intensity of the Raman peak. As described in reference [17,18], the longitudinal phonon characteristic length reduces as the defect density increases. The phonon were scattered by the lattice defect, impurity, and so on. So, the longitudinal mode is weaker as the characteristic length reduces and the lattice defect density increases.

Figure 2d shows the surface roughness image of the wafer. The Root-Mean-Square (RMS) of roughness is 5.6 nm, far greater than the GaAs single crystal wafer (< 1 nm). Thus, the surface roughness needs to be controlled while growing the epilayers. In this study, the epilayers were grown by the Molecular Beam Epitaxy (MBE) technique.

### 3.2. Optimization Using AlAs/GaAs Superlattice Structure

The AlAs/GaAs matching superlattice structure was used to optimize the substrate. As it is shown in Figure 1a, 10 nm AlAs with 10 nm GaAs epitaxy films were fabricated on the substrate with 10 periodicities, using MBE.

**Figure 3:**
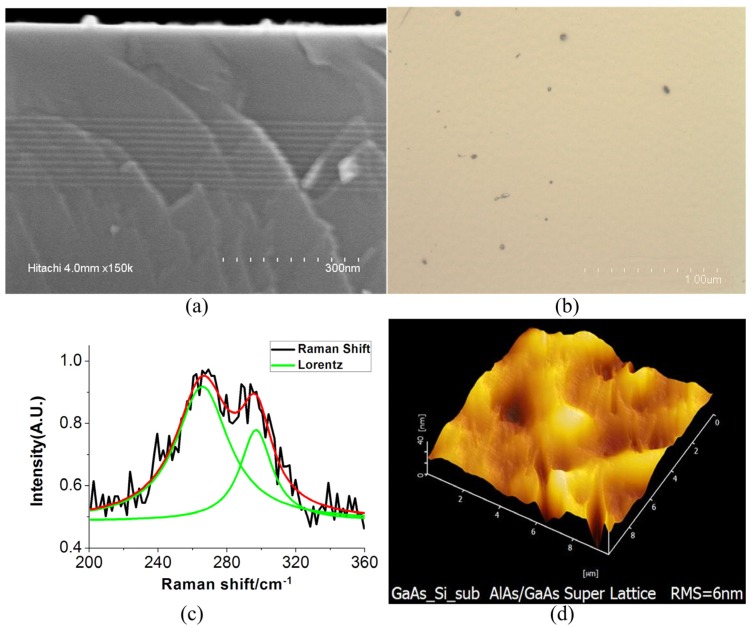
(**a**) The cross-section TEM image of optimized material; (**b**) the SEM image of EPD of optimized material; (**c**) Raman spectra measured of optimized material using Lorentz fit; and (**d**) the AFM image of optimized material.

With this method, we have characterized the improvement of the GaAs epitaxy layers, as it is shown in Figure 3. In Figure 3a, the dislocation lines have been bent by the superlattice structure and did not extend to the top layers. This means the matching superlattice assigns the mismatch strain to the layer and reduces the dislocation line and defects. This method can improve the quality of GaAs/Si wafers.

After the optimization by the matching superlattice structure AlAs/GaAs, the EPD was also characterized using FESEM, as it is shown in Figure 3b. The density of the lattice defect was calculated to be 1.41 × 10^5^ cm^−2^ and the matching superlattice structure has effectively assigned the mismatch strain to optimizing superlattice AlAs/GaAs layers. The matching superlattice AlAs/GaAs structure can reduce the mismatching strain and improve the quality of the material.

Figure 3c shows the Raman spectroscopy image of material, which has been optimized by matching the super lattice AlAs/GaAs structure. The curve has been done by the Lorentz fitting. In Figure 3c, the intensity of the peak is weak and the value is 265.72 cm^−1^, which has a red shift to the peak value 267 cm^−1^ of GaAs. The frequency shift value is −1.29 cm^−1^ and the FWHM is 37.61. This means that the residual stress in the optimized material is tensile stress, different from the stress in the wafer, which is compressive stress. The value of residual stress reduces to 739.45 MPa calculated with Equation (1). That means this wafer can be used as the novel substrate for MEMS sensors in the future. Meanwhile, the Raman spectrum is different with Figure 2c; there are two peaks in Figure 3c. Because of that, as described in reference [19,20], the phonon characteristic length reduces as the defect density increases. The transverse mode of phonon is an even parity and a Raman active mode, different from longitudinal mode. So, the longitudinal mode is weaker as the characteristic length reduces and the defect density increases.

In Figure 3c, there are two peaks. That means the longitudinal mode is stronger than the wafer (in Figure 2c). The longitudinal phonon spectroscopy becomes strong. That means the characteristic length of phonon become larger. More and more phonon can be excited in the Brillouin zone center [21]. The lattice defect in the material becomes less. The defect density of the wafer reduces with the SL structure. The quality of optimized material is better than the wafer. The result is consistent.

From the AFM image after the optimization, as it is shown in Figure 3d, the surface smoothness is 6nm. The MBE technique has efficiently controlled the roughness of the surface. The surface smoothness makes almost no change to the wafer.

### 3.3. Optimization Using InGaAs/GaAs Strain Superlattice Structure

The InGaAs/GaAs strain superlattice structure was used to optimize the substrate. As it is shown in Figure 1b, 20 nm InGaAs with 10 nm GaAs epitaxy films were fabricated on the substrate with 10 periodicities, and then 2nm GaAs were grown on stain superlattice as the buffer layer to grown matching super lattice AlAs/GaAs (shown in Figure 1b).

Strain superlattice structure grown on the epitaxy material layers will introduce a stress field in the whole material’s layers. This field can bend the dislocation lines, so that the dislocation lines cannot extend up to the epitaxy layers. At the same time, the lattice constant (0.605 nm) of InGaAs material is larger than the lattice constant (0.565 nm) of the GaAs material. It will produce the compressive Peach-Koehler force between the two material layers. This force can suppress the dislocation lines to extend from the strain source to reduce the defect in the epitaxy layers. This stress field can bend the dislocation lines parallel to the interface and cannot go up into the extend layers. It works as a dislocation filter [22,23].

**Figure 4:**
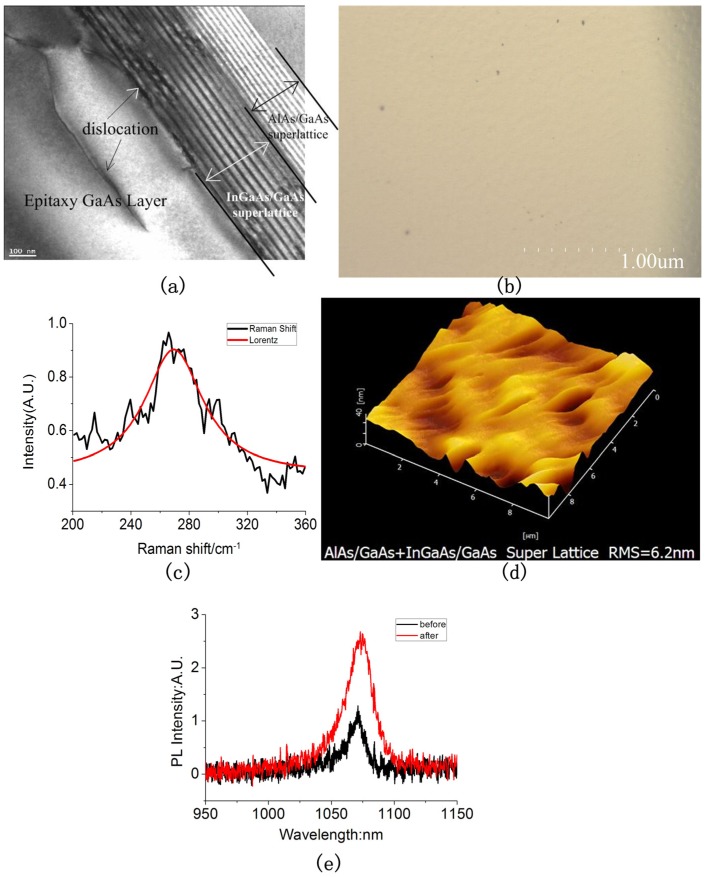
(**a**) The cross-section TEM image of material optimized by strain SL structure; (**b**) The SEM image of EPD after optimized material; (**c**) Raman spectra measured of optimized material using Lorentz fit; (**d**) The AFM image of optimized material; (**e**) The PL spectra measured of optimized material.

We optimized the wafer using the InGaAs/GaAs strain super lattice structure to study whether it can optimize the material. In order to ensure the consistency of the test results, we grow the InGaAs/GaAs strain superlattice structure on the optimized material (which is optimized by matching superlattice AlAs/GaAs structure) in our research. The results are shown in Figure 4:

From the TEM images as shown in Figure 4a, it can be concluded that the dislocation lines are filtered by the strain superlattice structure and cannot stretch upward. The results mean the superlattice structure can reduce the dislocation and defects. This is also further characterized quantitatively by FESEM, as it is shown in Figure 4b. The EPD in the image is reduced to 2.4 × 10^4^ cm^−2^. That means the strain superlattice has effectively bent the dislocation lines and the lines cannot extend up to epitaxy layers. The quality of material becomes better.

Figure 4c shows the Raman spectroscopy image after being optimized by the strain superlattice using Lorentz fitting. The image shows that the peak value of material after optimization is 269.49 cm^−1^. The peak of annealing material has a blue shift to the peak of GaAs. The frequency shift value is 2.49 cm^−1^, and the FWHM is 48.05. This means that the residual stress in the optimized material changes from tensile stress to compressive stress, and the value is 1.43 GPa. Because of the lattice constant (0.605 nm) of InGaAs material is larger than the lattice constant (0.565 nm) of GaAs material, when the InGaAs grows on the GaAs material, it will produce compressive stress in the GaAs material. So, the residual stress in the GaAs has changed to compressive stress after being optimized by the InGaAs/GaAs superlattice structure. From this, we can assure that the strain of supelattice structure can improve the quality of the material.

The surface roughness increases to 6.2 nm, as it is shown in Figure 4d. The roughness of the surface has been efficiently controlled the by MBE technique. The surface roughness is controlled to maintain the same level before and after growing the strain superlattice structure. This reduces the influence of surface roughness to the characteristics of luminescence of QW and makes the PL test results more acceptable.

Figure 4e shows the PL image before and after growing the strain superlattice on the matching superlattice material. The luminous intensity of QW after being optimized by the strain superlattice is twice as much as that optimized by the matching superlattice. This means the defect in the material and the surface roughness are both reduced. The reason is that the lattice defect as the non-radiation recombination centers can decrease the PL efficiency and reduce the luminous intensity. So, the lattice defect density reduces after being optimized by the superlattice structure. The superlattice structure has effectively bent the dislocation lines and makes inner stress in the control. So, the luminous intensity is larger than before, with the defect density reducing [24,25]. The result shows the quality of the material is improved. Thus, the strain superlattice can optimize the material.

The residual stress in the wafer is a key parameter as the substrate of MEMS sensors. It decides the mechanical strength of material and the reliability of sensors. The superlattice structure can also use its own stress to reduce the residual stress of the substrate material. In our study, the superlattice structure has reduced the residual stress in the material.

## 4. Conclusions

In this paper, the two kinds of superlattice structure have clearly improved the quality of the wafer. The defect density has reduced from 10^6^ cm^−1^ to 10^4^ cm^−1^, the residual stress reduces from the 1.57 GPa to 739.45 MPa and the surface roughness was controlled in a small float. So, the two superlattice structures are the effective methods to optimize the GaAs-on-Si material. It can be used to produce a novel substrate for MEMS sensors in the future.
